# Low-Molecular-Weight Fish Collagen Peptide (Valine-Glycine-Proline-Hydroxyproline-Glycine-Proline-Alanine-Glycine) Prevents Osteoarthritis Symptoms in Chondrocytes and Monoiodoacetate-Injected Rats

**DOI:** 10.3390/md21120608

**Published:** 2023-11-25

**Authors:** Wonhee Cho, Jeongjin Park, Jinhee Kim, Minhee Lee, So Jung Park, Kyung Seok Kim, Woojin Jun, Ok-Kyung Kim, Jeongmin Lee

**Affiliations:** 1 Department of Medical Nutrition, Kyung Hee University, Yongin 17104, Republic of Korea; wonhi1117@khu.ac.kr (W.C.); jinhee625@khu.ac.kr (J.K.); 2 Division of Food and Nutrition and Human Ecology Research Institute, Chonnam National University, Gwangju 61186, Republic of Korea; pjj8425@hanmail.net (J.P.); wjjun@jnu.ac.kr (W.J.); 3 Department of Food Innovation and Health, Kyung Hee University, Yongin 17104, Republic of Korea; miniclsrn@khu.ac.kr; 4 Suheung Co., Ltd., Seoul 02643, Republic of Korea; sjpark@suheung.com (S.J.P.); kskim1@suheung.com (K.S.K.)

**Keywords:** low-molecular-weight fish collagen, osteoarthritis, chondrocytes

## Abstract

The objective of this study was to investigate the effect of low-molecular-weight fish collagen (valine-glycine-proline-hydroxyproline-glycine-proline-alanine-glycine; LMWCP) on H_2_O_2_- or LPS-treated primary chondrocytes and monoiodoacetate (MIA)-induced osteoarthritis rat models. Our findings indicated that LMWCP treatment exhibited protective effects by preventing chondrocyte death and reducing matrix degradation in both H_2_O_2_-treated primary chondrocytes and cartilage tissue from MIA-induced osteoarthritis rats. This was achieved by increasing the levels of aggrecan, collagen type I, collagen type II, TIMP-1, and TIMP-3, while simultaneously decreasing catabolic factors such as phosphorylation of Smad, MMP-3, and MMP-13. Additionally, LMWCP treatment effectively suppressed the activation of inflammation and apoptosis pathways in both LPS-treated primary chondrocytes and cartilage tissue from MIA-induced osteoarthritis rats. These results suggest that LMWCP supplementation ameliorates the progression of osteoarthritis through its direct impact on inflammation and apoptosis in chondrocytes.

## 1. Introduction

Osteoarthritis, a degenerative joint disease, manifests as joint pain arising from a combination of cartilage degradation and synovial inflammation. This disease is a complex degenerative joint disorder characterized by the progressive deterioration of articular cartilage, changes in subchondral bone, and alterations in other joint tissues [1,2]. While the factors contributing to osteoarthritis remain unknown, it predominantly emerges after the age of 60. The pathogenesis of osteoarthritis is multifaceted and involves a combination of mechanical, biochemical, and genetic factors [3,4,5].

Osteoarthritis arises from an imbalance between the breakdown and repair of joint tissues, particularly the articular cartilage. The articular cartilage, a specialized connective tissue, serves to cushion and distribute load within the joint. This cartilage is composed of chondrocytes embedded within an organized extracellular matrix (ECM) of collagen and aggrecan [6,7]. In osteoarthritis, a chronic cycle of cartilage degradation and inadequate repair mechanisms leads to a net loss of the cartilage matrix. This process is driven by various molecular pathways, including the dysregulation of matrix metalloproteinases (MMPs), enzymes responsible for cartilage breakdown, and tissue inhibitors of metalloproteinases (TIMPs), which counteract MMP activity. Moreover, inflammation plays a key role in OA pathogenesis. Synovial inflammation, characterized by increased production of pro-inflammatory cytokines, chemokines, and mediators such as interleukin-1β (IL-1β), tumor necrosis factor-α (TNF-α), and prostaglandins, contributes to cartilage degradation and exacerbates joint damage. Additionally, activated immune cells and infiltrating macrophages further amplify the inflammatory response within the joint microenvironment [8,9,10].

Nonsteroidal anti-inflammatory drugs (NSAIDs) are frequently prescribed to alleviate the pain and inflammation associated with osteoarthritis. NSAIDs function by inhibiting cyclooxygenase (COX), thereby hindering the production of prostaglandins, which are crucial mediators in the inflammatory response. Nonetheless, NSAIDs are accompanied by gastrointestinal side effects [11,12]. Therefore, alternative remedies are often sought for the treatment of osteoarthritis due to their lower risk of side effects and minimal toxicity [13,14]. Collagen has recently garnered particular interest among researchers due to its multiple bioactive properties. However, the term “collagen” encompasses a variety of compounds with distinct structures, compositions, and origins, resulting in diverse properties and potential effects. Previous studies have proposed that collagen supplementation could promote the synthesis of connective tissue, especially cartilage ECM, mainly because collagen represents its major component. In fact, it has been demonstrated that certain peptides from hydrolyzed collagen are absorbed and accumulated in the cartilage [15,16,17]. Here, we investigated the effect of low-molecular-weight fish-derived type I collagen hydrolysate (LMWCP; valine-glycine-proline-hydroxyproline-glycine-proline-alanine-glycine), originating from tilapia, in primary chondrocytes and rats with monosodium iodoacetate (MIA)-induced osteoarthritis to identify mechanisms underlying the cartilage-regenerating properties of collagen.

## 2. Results

### 2.1. LMWCP Ameliorated Cell Damage and the Expression of Catabolic Factors in H_2_O_2_-Treated Chondrocytes

**Figure 1:**
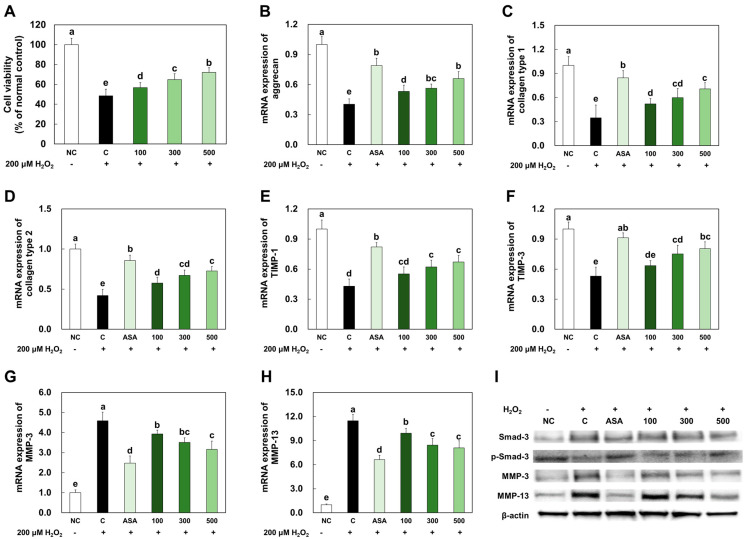
Effects of LMWCP on cell viability (**A**), mRNA expression of aggrecan (**B**), collagen type 1 (**C**), collagen type 2 (**D**), TIMP-1 (**E**), TIMP-3 (**F**), MMP-3 (**G**), MMP-13 (**H**), and protein expression of p-Smad-3, MMP-3, and MMP-13 (**I**) in H_2_O_2_-treated chondrocytes. NC: no treatment, C: 200 μM of H_2_O_2_ treatment, ASA: 200 μM of H_2_O_2_ and 10 μM acetylsalicylic acid, 100: 200 μM of H_2_O_2_ and 100 μg/mL of LMWCP, 300: 200 μM of H_2_O_2_ and 300 μg/mL of LMWCP, 500: 200 μM of H_2_O_2_ and 500 μg/mL of LMWCP. Values are presented as mean ± SD. Different letters (a > b > c > d > e) indicate a significant difference at *p* < 0.05 according to Duncan’s multiple range test.

LMWCP pretreatment ameliorated 200 μM H_2_O_2_-induced cell death (Figure 1A). The mRNA expression levels of anabolic factors, such as aggrecan, collagen type I, collagen type II, TIMP-1, and TIMP-3, were significantly decreased in the H_2_O_2_-treated chondrocytes compared to those in the normal control (NC). However, acetylsalicylic acid (PC) or LMWCP treatment significantly increased mRNA expression levels of these anabolic factors in the H_2_O_2_-treated chondrocytes (*p* < 0.05; Figure 1B–F). mRNA expression levels of catabolic factors, including MMP-3 and MMP-13, were significantly increased in the H_2_O_2_-treated chondrocytes compared to those in NC, while acetylsalicylic acid or LMWCP treatment significantly increased mRNA expression levels of these catabolic factors in the H_2_O_2_-treated chondrocytes (*p* < 0.05; Figure 1G,H).

The protein expression levels of catabolic factors, such as Smad3 phosphorylation, MMP-3, and MMP-9, in the H_2_O_2_-treated chondrocytes were investigated. The protein expression level of Smad3 phosphorylation was significantly decreased in the H_2_O_2_-treated chondrocytes compared to those in NC, but acetylsalicylic acid or LMWCP treatment significantly increased protein expression in the H_2_O_2_-treated chondrocytes. The protein expression levels of MMP-3 and MMP-9 were increased in the H_2_O_2_-treated chondrocytes compared to those in NC. However, acetylsalicylic acid or LMWCP treatment decreased the protein expression levels in the H_2_O_2_-treated SW982 cells (Figure 1I).

### 2.2. LMWCP Ameliorated Inflammation in LPS-Treated Chondrocytes

**Figure 2:**
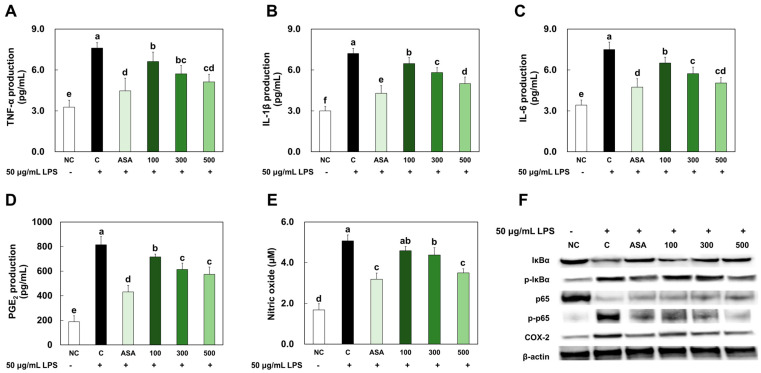
Effects of LMWCP on the production of TNF-α (**A**), IL-1β (**B**), and IL-6 (**C**), PGE2 (**D**), and nitric oxide (**E**) and the protein expression of p-IκBα, p-p65, and COX-2 (**F**) in LPS-treated chondrocytes. NC: no treatment, C: 200 μM of H_2_O_2_ treatment, ASA: 200 μM of H_2_O_2_ and 10 μM acetylsalicylic acid, 100: 200 μM of H_2_O_2_ and 100 μg/mL of LMWCP, 300: 200 μM of H_2_O_2_ and 300 μg/mL of LMWCP, 500: 200 μM of H_2_O_2_ and 500 μg/mL of LMWCP. Values are presented as mean ± SD. Different letters (a > b > c > d > e > f) indicate a significant difference at *p* < 0.05 by Duncan’s multiple range test.

The levels of pro-inflammatory cytokines, prostaglandin (PGE) 2, and NO were significantly increased in the LPS-treated chondrocytes compared with those in NC. However, acetylsalicylic acid or LMWCP treatment significantly decreased the levels of those factors in the LPS-treated chondrocytes (*p* < 0.05; Figure 2A–E). The protein expression levels of phospho-IκBα/IκBα, phospho-p65/p65, and COX-2 were significantly increased in the LPS-treated chondrocytes compared with those in NC, whereas acetylsalicylic acid or LMWCP treatment significantly decreased the levels of those factors in the LPS-treated chondrocytes (Figure 2F).

### 2.3. LMWCP Ameliorated Apoptosis in LPS-Treated Chondrocytes

**Figure 3:**
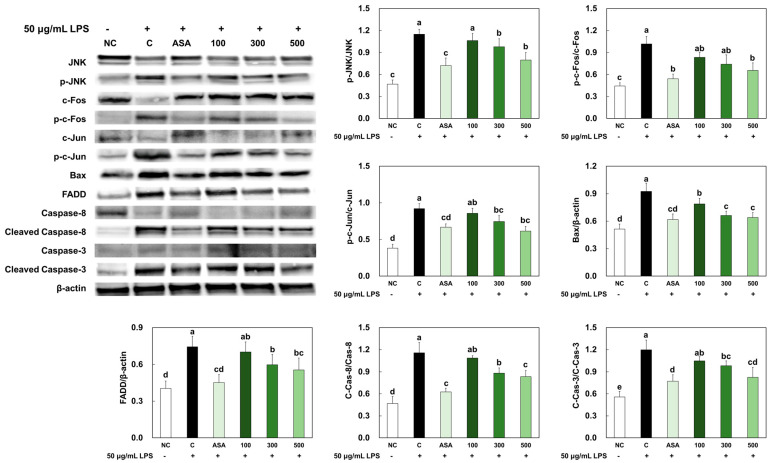
Effects of LMWCP on protein expression of apoptosis factors in LPS-treated chondrocytes. NC: no treatment, C: 200 μM of H_2_O_2_ treatment, ASA: 200 μM of H_2_O_2_ and 10 μM acetylsalicylic acid, 100: 200 μM of H_2_O_2_ and 100 μg/mL of LMWCP, 300: 200 μM of H_2_O_2_ and 300 μg/mL of LMWCP, 500: 200 μM of H_2_O_2_ and 500 μg/mL of LMWCP. Values are presented as mean ± SD. Different letters (a > b > c > d > e) indicate a significant difference at *p* < 0.05 by Duncan’s multiple range test.

As illustrated in Figure 3, LPS treatment stimulated apoptosis signaling pathways, including the c-Jun N-terminal kinase (JNK)/c-Fos and c-Jun pathway, as well as the Fas-associated protein with death domain (FADD)/caspase8/Bax/caspase3 pathway in the primary chondrocytes. In the primary chondrocytes treated with acetylsalicylic acid or LMWCP, the expression of proteins involved in the apoptosis pathways was suppressed compared to those without treatment.

### 2.4. LMWCP Ameliorated Morphological Change of Cartilage Tissue in MIA-Injected Rats

**Figure 4:**
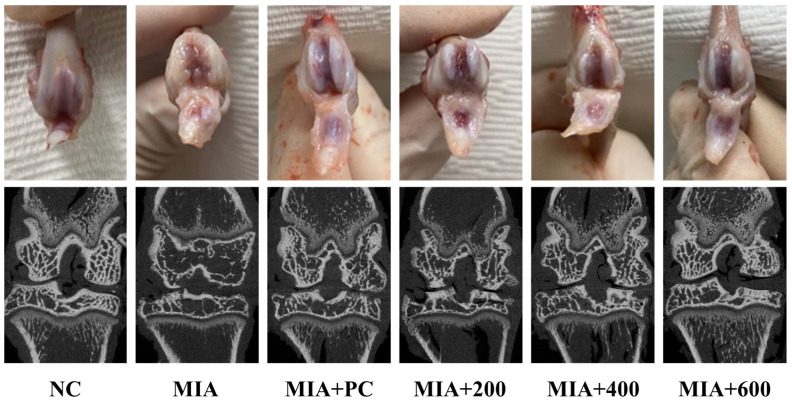
Effects of LMWCP on the morphological features of cartilage tissue from MIA-induced osteoarthritis rats. NC: saline injection, MIA: MIA injection, MIA + PC: MIA injection and 50 mg/kg BW of ibuprofen, MIA + 200: MIA injection and 200 mg/kg BW of LMWCP, MIA + 400: MIA injection and 400 mg/kg BW of LMWCP, MIA + 600: MIA injection and 600 mg/kg BW of LMWCP.

In the cartilage tissue of MIA-induced osteoarthritis rats, the observed morphological changes were characterized by a noticeable loss of luster and the presence of cartilage fibrillation and erosion. Additionally, MIA-induced osteoarthritis rats exhibited an irregular articular cartilage surface and cartilage matrix degradation, while the joints of non-induced osteoarthritis rats possessed a smooth articular cartilage surface. In contrast, the group treated with ibuprofen or LMWCP exhibited suppressed and morphological changes in the cartilage tissue and displayed an increase in cartilage surface volumes. This suggests that treatment with ibuprofen or LMWCP may have a protective effect on the integrity and structure of the cartilage tissue, potentially mitigating the degenerative changes associated with osteoarthritis (Figure 4).

### 2.5. LMWCP Ameliorated Catabolic Factors Expression in Cartilage Tissue from MIA-Injected Rats

**Figure 5:**
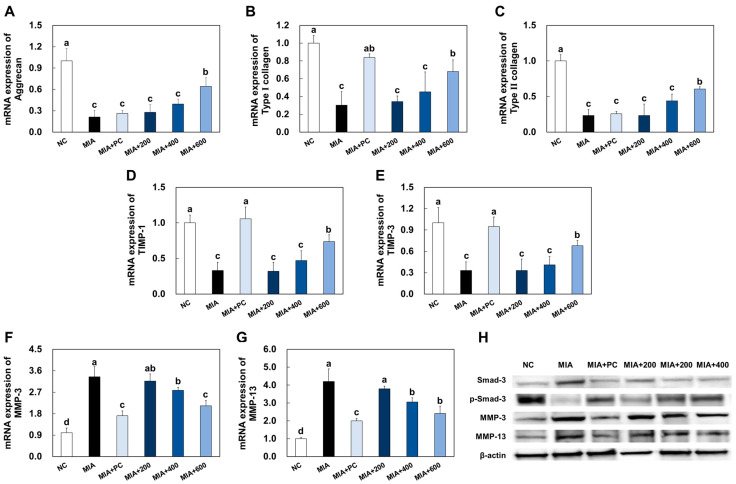
Effects of LMWCP on mRNA expression of aggrecan (**A**), collagen type 1 (**B**), collagen type 2 (**C**), TIMP-1 (**D**), TIMP-3 (**E**), MMP-3 (**F**), MMP-13 (**G**), and protein expression of p-Smad-3, MMP-3, and MMP-13 (**H**) in cartilage tissue from MIA-induced osteoarthritis rats. NC: saline injection, MIA: MIA injection, MIA + PC: MIA injection and 50 mg/kg BW of ibuprofen, MIA + 200: MIA injection and 200 mg/kg BW of LMWCP, MIA + 400: MIA injection and 400 mg/kg BW of LMWCP, MIA + 600: MIA injection and 600 mg/kg BW of LMWCP. All values are presented as mean ± SD. Different letters (a > b > c > d) indicate a significant difference at *p* < 0.05 according to Duncan’s multiple range test.

The mRNA expression levels of the anabolic factors, aggrecan, collagen type I, collagen type II, TIMP-1, and TIMP-3, were significantly decreased in cartilage tissue from MIA-injected rats compared to those in normal rats (NC). However, mRNA expression levels of the anabolic factors were significantly increased in groups supplemented with ibuprofen or LMWCP compared to the group with MIA-induced osteoarthritis (*p* < 0.05; Figure 5A–E).

The protein expression level of Smad3 phosphorylation was significantly decreased, and MMP-3 and MMP-9 were increased in cartilage tissue from MIA-injected rats compared to those in normal control. However, ibuprofen or LMWCP supplementation significantly increased Smad3 phosphorylation and decreased MMP-3 and MMP-9 in cartilage tissue from MIA-injected rats compared to those in the MIA injection control (*p* < 0.05; Figure 5F,G).

### 2.6. LMWCP Ameliorated Inflammation in Cartilage Tissue from MIA-Injected Rats

**Figure 6:**
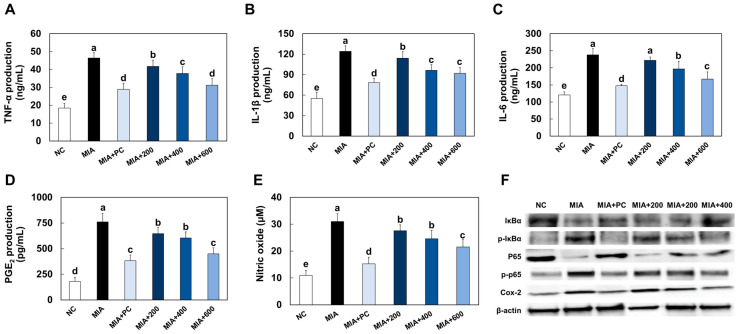
Effects of LMWCP on serum levels of TNF-α (**A**), IL-1β (**B**), and IL-6 (**C**), PGE2 (**D**), and nitric oxide (**E**) and protein expression of p-IκBα, p-p65, and COX-2 (**F**) in cartilage tissue from MIA-induced osteoarthritis rats. NC: saline injection, MIA: MIA injection, MIA + PC: MIA injection and 50 mg/kg BW of ibuprofen, MIA + 200: MIA injection and 200 mg/kg BW of LMWCP, MIA + 400: MIA injection and 400 mg/kg BW of LMWCP, MIA + 600: MIA injection and 600 mg/kg BW of LMWCP. Values are presented as mean ± SD. Different letters (a > b > c > d > e) indicate a significant difference at *p* < 0.05 by Duncan’s multiple range test.

MIA injection increased the production of pro-inflammatory cytokines (TNF-α, IL-1β, and IL-6), PGE2, and NO in rats. However, ibuprofen or LMWCP supplementation significantly decreased the levels of these factors in cartilage tissue from MIA-induced osteoarthritis rats (*p* < 0.05; Figure 6A–E). MIA injection increased the protein expression levels of phospho-IκBα/IκBα, phospho-p65/p65, and COX-2 in cartilage tissue. However, ibuprofen or LMWCP treatment significantly decreased the levels of these factors in the LPS-treated chondrocytes (Figure 6F).

### 2.7. LMWCP Ameliorated Apoptosis in Cartilage Tissue from MIA-Injected Rats

**Figure 7:**
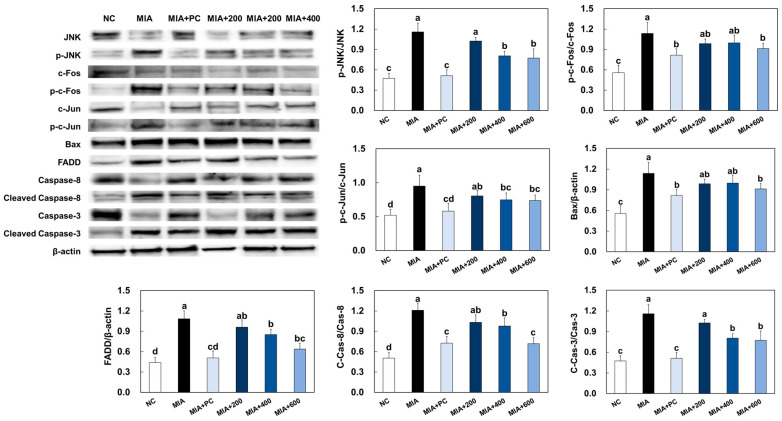
Effects of LMWCP on protein expression of apoptosis factors in cartilage tissue from MIA-induced osteoarthritis rats. NC: saline injection, MIA: MIA injection, MIA + PC: MIA injection and 50 mg/kg BW of ibuprofen, MIA + 200: MIA injection and 200 mg/kg BW of LMWCP, MIA + 400: MIA injection and 400 mg/kg BW of LMWCP, MIA + 600: MIA injection and 600 mg/kg BW of LMWCP. Values are presented as mean ± SD. Different letters (a > b > c > d) indicate a significant difference at *p* < 0.05 by Duncan’s multiple range test.

MIA injection stimulated apoptosis signaling pathways, including the JNK/c-Fos and c-Jun pathway and the FADD/caspase8/Bax/caspase3 pathway, in cartilage tissue from rats. However, ibuprofen or LMWCP supplementation significantly suppressed these apoptosis signaling pathways in cartilage tissue from MIA-induced osteoarthritis rats (*p* < 0.05; Figure 7).

## 3. Discussion

Various studies have investigated whether the intake of food-derived collagen influences collagen production in the body [15,16,17,18,19]. For example, Iwai et al. identified changes in the levels of collagen peptides in human blood after the oral ingestion of gelatin hydrolysates. The authors demonstrated that collagen-derived dipeptides, such as Pro-Hyp, and tripeptides, such as Pro-Hyp-Gly, were detected in the systemic blood within an hour after ingestion [18]. Yazaki et al. also investigated the levels of collagen-derived peptides in the blood after the ingestion of high tripeptide-containing collagen hydrolysate in humans and transiently identified 17 types of collagen-derived peptides, with a particular enrichment in Gly-Pro-Hyp. Additionally, the authors detected a higher enrichment of Pro-Hyp in the plasma and skin, derived from Gly-Pro-Hyp hydrolysis, upon administration of Gly-Pro-Hyp peptide in a mouse model [19]. These data suggest that dietary collagen affects the body’s collagen composition. Therefore, our study aimed to investigate the inhibitory effect of LMWCP on osteoarthritis and compare it with positive controls using ibuprofen or acetylsalicylic acid.

Osteoarthritis is characterized by the progressive deterioration of articular cartilage, primarily driven by MMPs and inflammatory substances. Collagen, which constitutes approximately 30% of the body’s total protein, forms the backbone of the extracellular matrix. Another vital component is aggrecan, a large proteoglycan that is indispensable for normal joint function. The overall quality of the extracellular matrix is crucial in maintaining the functional integrity of cartilage. Specifically, MMPs, known as collagenases, play a pivotal role in collagen breakdown, making them the primary mediators of this process. MMP-3, also known as stromelysins, primarily target non-collagen matrix proteins, whereas MMP-13, referred to as collagenases, primarily target interstitial collagens such as types I, II, and III. The activity of these MMPs is intricately regulated by TIMPs (tissue inhibitors of metalloproteinases) [20,21,22,23]. In our study, we observed an increase in MMPs, which was accompanied by a decrease in the mRNA expression levels of key components including aggrecan, collagen, and TIMPs, within the cartilage tissue of rats with MIA-induced osteoarthritis. However, following the administration of LMWCP, we noted a suppression of these factor changes in the cartilage tissue of rats with MIA-induced osteoarthritis. This suggests that LMWCP effectively inhibits the degradation of ECM (extracellular matrix) within the cartilage tissue.

Inflammation is a crucial factor in the development and progression of osteoarthritis. The mechanism of osteoarthritis, particularly in relation to inflammation, involves various elements, with a significant role played by the activation of nuclear factor-kappa B (NF-κB) and subsequent production of inflammatory cytokines. NF-κB is a protein complex that serves as a key regulator of immune and inflammatory responses. In osteoarthritis, NF-κB is frequently activated, often in response to factors like cytokines, including IL-1β and TNF-α, which stimulate the release of MMPs and other catabolic enzymes, thereby exacerbating osteoarthritis [24]. In our study, we have demonstrated that treatment with LMWCP effectively suppressed the phosphorylation of IκB and p65, as well as the production of inflammatory mediators in both the LPS-treated primary chondrocytes and the cartilage tissue of rats with MIA-induced osteoarthritis. These findings strongly suggest that LMWCP treatment provides a protective effect against inflammation in chondrocytes by inhibiting the NF-κB signaling pathways.

In the development of osteoarthritis, the molecular pathways that regulate chondrocyte apoptosis are influenced by various factors, including the activation of signaling cascades [25,26]. Two significant pathways implicated in this process are the JNK/c-Fos and c-Jun pathway, as well as the FADD/caspase8/Bax/caspase3 pathway. The JNK pathway is activated in response to various stress stimuli, including inflammation and oxidative stress, which are prevalent in osteoarthritis. This pathway leads to the activation of transcription factors such as c-Fos and c-Jun, which regulate the expression of genes involved in cell proliferation, differentiation, and apoptosis. In the context of osteoarthritis, the activation of JNK and subsequent upregulation of c-Fos and c-Jun can lead to increased apoptotic signaling in chondrocytes, contributing to the degradation of articular cartilage. Additionally, the FADD pathway is a key pathway in the extrinsic apoptotic pathway activated by pro-inflammatory cytokines and oxidative stress in the osteoarthritic state. This pathway leads to the recruitment and activation of caspase-8, initiating a caspase cascade, which in turn activates downstream caspases, including caspase-3, a pivotal executioner caspase in apoptosis. These pathways collectively contribute to the initiation and execution of apoptosis in chondrocytes, ultimately leading to the degradation of articular cartilage observed in osteoarthritis [25,26,27].

Here, we demonstrated that LMWCP treatment suppressed the activation of the apoptosis pathway in both the LPS-treated primary chondrocytes and the cartilage tissue of rats with MIA-induced osteoarthritis. Therefore, our findings suggest that LMWCP supplementation can prevent the development of osteoarthritis through the suppression of apoptosis in chondrocytes. Although this study confirmed that LMWCP had a positive effect on joint health, it was not confirmed whether the ingested LMWCP was directly utilized for cartilage production, and therefore, additional studies are needed to clarify this.

## 4. Materials and Methods

### 4.1. Preparation of LMWCP

The collagen used in this study was purified from fish scale collagen originating from tilapia (Oreochromis genus) gelatin, which was sourced from GELTECH in Busan, Korea. First, the dried tilapia scale was treated with HCl and then extracted with hot water. The extract was further purified by filtering and ion exchange. Then, the purified material was concentrated and sterilized. The resulting extract was heat-dried and milled, ready as gelatin for the next collagen production. Collagen production was initiated by melting the gelatin, followed by treatment with protease. Then, the solution was deodorized by treating it in an activated carbon tower. Subsequently, the gelatin was purified multiple times using pulp, carbon, and cartridge filters. The resulting crude peptides underwent high-temperature treatment (120 ± 5 °C) for 10–20 s to ensure sterilization. The products were then spray-dried at 170–230 °C and 150–230 bar, followed by sieving to achieve an average particle size of 50–150 μm. The low-molecular-weight fish collagen contained 0.93 mg/g of octapeptide (valine-glycine-proline-hydroxyproline-glycine-proline-alanine-glycine; LMWCP), with an average molecular weight of 667.7 Da.

### 4.2. Primary Culture of Chondrocytes

Two Sprague-Dawley rats (6 weeks old, male) were obtained from SaeRon Bio in Uiwang, Korea, and were humanely euthanized by cervical dislocation. Cartilages were then isolated and incubated overnight in Hank’s balanced salt solution (Hyclone Laboratories, Logan, UT, USA) containing 2 mg/mL collagenase (C0130, Sigma-Aldrich Co, St. Louis, MO, USA), and overnight in a shaker at 100 rpm. The obtained chondrocytes were seeded in 75T flasks and cultured in Dulbecco’s minimal essential medium (Hyclone Laboratories) supplemented with 10% fetal bovine serum (Hyclone Laboratories), 100 mg/L penicillin-streptomycin (Hyclone Laboratories), and 2 mmol/L glutamine (Hyclone Laboratories). The cultures were maintained at 37 °C in a humid atmosphere with 5% CO_2_. We performed medium replacement every three days, initiating subculturing when the culture reached approximately 80% confluence. Only cells with a passage number not exceeding 10 were employed for experimental purposes.

### 4.3. Animals and Induction of Osteoarthritis

**Figure 8:**
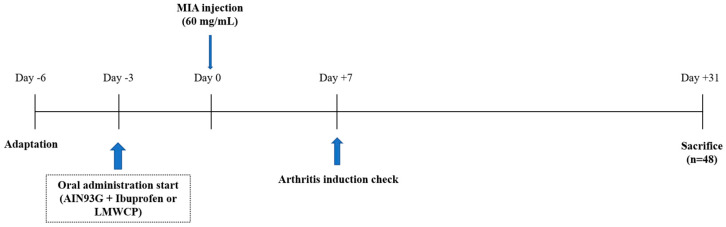
The experimental schedule.

Sprague-Dawley rats (6 weeks old, male) were obtained from SaeRon Bio in Uiwang, Korea. These rats were acclimated in a controlled environment room, with conditions set at 23 ± 2 °C, a 12/12 h light/dark cycle, and a relative humidity of 50 ± 5%. Ethical approval for the experiments was granted by the Institutional Animal Care and Use Committee of Kyung Hee University (KHGASP-22-251). Prior to initiating the experiment, the rats were housed in cages for one week to acclimate to their surroundings. To induce osteoarthritis, anesthesia was administered to all animals using a consistent concentration of isoflurane, and 60 mg/mL of MIA was injected into the articular space of the knee joints. The rats were divided into six groups, each consisting of eight rats, as follows: normal control (NC), 50 μL of saline was injected into both the left/right knee joint + AIN93G; MIA, 50 μL of MIA (60 mg/mL) was injected into both the left/right knee joint + AIN93G; MIA + PC, 50 μL of MIA (60 mg/mL) was injected into both the left/right knee joint + ibuprofen 20 mg/kg body weight (BW) in AIN93G; MIA + 200, 50 μL of MIA (60 mg/mL) was injected into both the left/right knee joint + LMWCP 200 mg/kg BW in AIN93G; MIA + 400, 50 μL of MIA (60 mg/mL) was injected into both the left/right knee joint + LMWCP 400 mg/kg BW in AIN93G; MIA + 600, 50 μL of MIA (60 mg/mL) was injected into both the left/right knee joint + LMWCP 600 mg/kg BW in AIN93G (Table 1). The rats were monitored for clinical signs of osteoarthritis on a weekly basis following the MIA injection, clinical signs were assessed by observing swelling in the knee joint of rats to determine the presence or absence of inflammation. After completion of the experiment (Figure 8), rats were anesthetized with isoflurane, followed by blood collection through the abdominal vena cava. Subsequently, the hearts were excised and euthanized, and both blood and knee joint tissues were stored at −70 °C until each experiment.

### 4.4. Micro-CT and ELISA Analysis

Micro-CT imaging of the formalin-fixed articular cartilage from rats was used to measure the roughness of the bone surface. Micro-CT image scanning was conducted using the Skyscan 1172^®^ X-ray μCT scanning system (Bruker, Belgium). After standardized reconstruction of the scanned images, the data for each sample were obtained using the micro-CT software to orient each sample in the same manner. The levels of inflammatory cytokines (TNF-α, IL-1β, and IL-6), PGE_2_, and nitric oxide were measured using an ELISA kit (R&D Systems, Minneapolis, MN, USA) according to the manufacturer’s instructions.

### 4.5. Total RNA Extraction and Real-Time PCR

Total RNA from chondrocytes and cartilage tissue was extracted using a commercial RNA extraction kit (QIAGEN, Gaithersburg, MD, USA), and the extracted RNA was assessed for ratio and concentration using Nano-drop. The cDNA was synthesized from purified total RNA (500 ng/mL) using the iScript^TM^ cDNA Synthesis kit (Bio-Rad, Hercules, CA, USA). Real-time PCR was conducted using a CFX Connect^TM^ Real Time System (Bio-Rad) with the iScript^TM^ Green Supermix, cDNA, and custom designed primers (Table 2) and the real-time PCR reactions run in duplicates. Data analysis was conducted using the CFX Manager^TM^ 3.1 analysis software (Bio-Rad).

### 4.6. Western Blot

Total protein from chondrocytes and cartilage tissue was extracted using 4X NuPAGE LDS sample buffer (Life Technologies, Gaithersburg, MD, USA). Protein samples containing 20~100 μg of protein from cells were separated by gel electrophoresis and transferred onto membranes. The membranes were blocked, followed by incubation with primary antibodies (Smad-3, p-Smad-3, MMP-3, MMP-13, IκBα, p-IκBα, p65, p-p65, COX-2, JNK, p-JNK, c-Fos, p-c-Fos, c-Jun, p-c-Jun, Bax, FADD, caspase-8, cleaved caspase-8, caspase-3, cleaved caspase-3, and β-actin and secondary antibodies (Table 3). Bands were captured using the ChemiDoc Imaging System (Bio-Rad, Hercules, CA, USA) and quantified with ImageJ software version 1.53e (National Institutes of Health, Bethesda, MD, USA).

### 4.7. Statistical Analysis

The data were reported as mean (*n* = 3 or 8) ± standard deviation (SD). Statistical analyses were conducted using Duncan’s multiple range tests following a one-way analysis of variance. All *p*-values < 0.05 were considered statistically significant, and the statistical analyses were carried out using SPSS software (SPSS PASW Statistic v.23.0, SPSS Inc., Chicago, IL, USA).

## 5. Conclusions

We demonstrated that LMWCP ameliorated osteoarthritis symptoms, including inflammation and degradation of articular cartilage in primary chondrocytes and rats with MIA-induced osteoarthritis. Our findings demonstrated that LMWCP supplementation has a beneficial effect in the treatment of osteoarthritis, as it suppresses the expression of catabolic factors. Moreover, LMWCP inhibited inflammation and apoptosis in both the LPS-treated primary chondrocytes and the cartilage tissue of rats with MIA-induced osteoarthritis. The study not only provides scientific evidence of the cartilage-regenerating effects of LMWCP but also offers insights into the mechanisms responsible for the anti-inflammatory and anti-osteoarthritis properties of LMWCP.

## Figures and Tables

**Table 1:** Experimental groups (*n* = 8).

Group	Diet	Supplement	MIA
Normal control	AIN93G	−	−
MIA	AIN93G	−	+
MIA + PC	AIN93G	Ibuprofen 20 mg/kg BW	+
MIA + 200	AIN93G	LMWCP 200 mg/kg BW	+
MIA + 400	AIN93G	LMWCP 400 mg/kg BW	+
MIA + 600	AIN93G	LMWCP 600 mg/kg BW	+

**Table 2:** Primer sets used for real-time RT-PCR.

Gene	Forward Sequence (5′-3′)	Reverse Sequence (5′-3′)
GAPDH (NM_017008)	TGG CCT CCA AGG AGT AAG AAA C	CAG CAA CTG AGG GCC TCT CT
Aggrecan (NM_022190)	GAA GTG GCG TCC AAA CCA A	CGT TCC ATT CAC CCC TCT CA
Collagen Type I (NM_000088)	GAG CGG AGA GTA CTG GAT CGA	CTG ACC TGT CTC CAT GTT GCA
Collagen Type II (RATCOLLII)	GCA ACA GCA GGT TCA CGT ACA	TCG GTA CTC GAT GAT GGT CTT G
TIMP-1 (NM_053819)	ACA GCT TTC TGC AAC TCG GA	CGT CGA ATC CTT TGA GCA TC
TIMP-3 (RNU27201)	CTT CTG CAA CTC CGA CAT CGT	GGG GCA TCT TAC TGA ATC CTC
MMP-3 (NM_133523)	GAG TGT GGA TTC TGC CAT TGA G	TTA TGT CAG CCT CTC CTT CAG AGA
MMP-13 (NM_133530.1)	ACG TTC AAG GAA TCC AGT CT	GGA TAG GGC TGG GTC ACA CTT

**Table 3:** Antibodies used for Western blot analysis.

Antibodies	Distributor
Smad3	Cell signaling (#9523, Beverly, MA, USA)
p-Smad3	Cell signaling (#9520)
MMP-3	Abcam (ab53015)
MMP-13	Abcam (ab39012)
IκBα	Cell signaling (#9242)
p-IκBα	Cell signaling (#2859)
p65	Abcam (ab16502)
p-p65	Cell signaling (#3031)
COX-2	Cell signaling (#12282)
JNK	Cell signaling (#9252)
p-JNK	Cell signaling (#9251)
c-Fos	Cell signaling (#4384
p-c-Fos	Cell signaling (#5348)
c-Jun	Cell signaling (#9165)
p-c-Jun	Cell signaling (#2361)
Bax	Cell signaling (#2772)
FADD	LSbio (LS-C766496, Settle, WA, USA)
Caspase-8	Cell signaling (#4790)
Cleaved caspase-8	Cell signaling (#8592)
Caspase-3	Cell signaling (#9662)
Cleaved caspase-3	Cell signaling (#9661)
β-actin	BETHYL (A300-491A, Waltham, MA, USA)

MMP, matrix metallopeptidase; COX-2, cyclooxygenase-2; JNK, c-Jun N-terminal kinase; Host animal, Rabbit; Dilution for western blot, 1:1000.

## Data Availability

Data are contained within the article.
